# Scabicidal Potential of Coconut Seed Extract in Rabbits via Downregulating Inflammatory/Immune Cross Talk: A Comprehensive Phytochemical/GC-MS and In Silico Proof

**DOI:** 10.3390/antibiotics12010043

**Published:** 2022-12-27

**Authors:** Eman Maher Zahran, Nehad M. Reda Abdel-Maqsoud, Omar. Y. Tammam, Islam M. Abdel-Rahman, Mahmoud A. Elrehany, Hussain T. Bakhsh, Faisal H. Altemani, Naseh A. Algehainy, Mubarak A. Alzubaidi, Usama Ramadan Abdelmohsen, Abeer H. Elmaidomy

**Affiliations:** 1 Department of Pharmacognosy, Faculty of Pharmacy, Deraya University, Minia 61111, Egypt; 2 Department of Pathology, Faculty of Pharmacy, Deraya University, Minia 61111, Egypt; 3 Department of Pathology, Faculty of Medicine, Minia University, Minia 61519, Egypt; 4 Department of Biochemistry, Faculty of Pharmacy, Deraya University, Minia 61111, Egypt; 5 Department of Pharmaceutical Chemistry, Faculty of Pharmacy, Deraya University, Minia 61111, Egypt; 6 Department of Pharmacy Practice, Faculty of Pharmacy, King Abdulaziz University, Jeddah 21589, Saudi Arabia; 7 Department of Medical Laboratory Technology, Faculty of Applied Medical Sciences, University of Tabuk, Tabuk 71491, Saudi Arabia; 8 Department of Biological Sciences, Faculty of Science, King Abdulaziz University, Jeddah 21589, Saudi Arabia; 9 Department of Pharmacognosy, Faculty of Pharmacy, Minia University, Minia 61519, Egypt; 10 Department of Pharmacognosy, Faculty of Pharmacy, Beni-Suef University, Beni-Suef 62514, Egypt

**Keywords:** coconut seed extract, *Sarcoptes scabiei*, scabies, GC-MS, IL-1β, IL-6, IL-10

## Abstract

Scabies is an invasive skin condition caused by *Sarcoptes scabiei* mites. The present study investigates the antiscabies potential of coconut seed extract (CSE) in rabbits. GC-MS analysis of the seed oil identified 17 known compounds, while CSE phytochemical investigation afforded 4 known ones. The topical application of seed extract improved all signs of infection, and the improvement started 3 days post application. However, in vitro application of the extract caused 99% mortality of mites 1 day post application. Histopathological examination revealed the absence of inflammatory infiltration and hyperkeratosis of the epidermis, compared with ivermectin-treated groups which revealed less improvement. The mRNA gene expression results revealed a suppression of IL-1β, IL-6, IL-10, MMP-9, VEGF, and MCP-1, and an upregulation of I-CAM-1, KGF as well as TIMP-1. The docking analysis emphasized a strong binding of gondoic acid with IL-1β, IL-6, and VEGF with high binding scores of −5.817, −5.291, and −8.362 kcal/mol, respectively, and a high binding affinity of 3″(1‴-*O*-*β*-D-glucopyranosyl)-sucrose with GST with −7.24 kcal/mol. Accordingly, and for the first time, our results highlighted the scabicidal potential of coconut seed extract, which opens the gate for an efficient, cost-effective as well as herbal-based alternative for the control of scabies in rabbits.

## 1. Introduction

Natural products have been widely used as a rich source for antimicrobials with structural as well as chemical diversity for the purpose of drug discovery. Since antimicrobial resistance increases for market antibiotics once they are used in clinical practice, the need for nature-based alternatives has increased [1,2].

The scabies itchy mite, *Sarcoptes scabiei*, is a medically important pathogenic ectoparasite of human as well as other mammals worldwide [3]. The mites burrow deep inside the stratum corneum layer resulting in inflammation, immunity reactions, and progressive skin hyper-keratinization, usually followed by bacterial infections or renal/cardiac ailments [4]. Scabies was added to the World Health Organization (WHO) list of neglected tropical diseases in 2017, which highlights the substantial global health and economic burden, especially in low income populations [5]. Similar to human infection, rabbits are the most affected animals by *Sarcoptes* infection, known as ‘mange’ which results in economic losses of rabbit colonies, reduction in their productivity, and lethality in the case of absence of treatment [3]. Mange in rabbits is usually treated with synthetic acaricides such as ivermectin, doramectin, organophosphates, and pyrethroids, which have serious side effects and environmental hazardous impacts [6,7,8,9].

Mites exert a long co-evolution with their hosts and stimulate the host’s keratinocytes with their eggs, feces, saliva, and enzymes resulting in inflammation [10]. Later, they stimulate fibroblasts, endothelial cells, and immune effector cells including mast cells, macrophages, and lymphocytes which carry antigens from the mites to the lymphatic tissue to start the adaptive immune responses [11]. As a result, a cross talk occurs between inflammatory and anti-inflammatory cytokines as well as chemokines, which results in the appearance of scabies symptoms [11].

The synthetic acaricides mainly work on mange mites nerve axons by modifying the kinetics of ligand-gated ion channels of mange mites and breaking their life cycles [6,7,12,13]. Disadvantages include drug resistance, treatment delay due to the absence of a significant effect on tissue healing, environmental toxicity, and high cost especially for resource-poor farmers who extensively deal with mammals in the developing world [14]. Natural products are an excellent alternative to synthetic drugs due to the richness of bio-actives that have been widely used for fighting against parasitic infections [11]. Such phytoconstituents attain broad spectrum efficacies, multiple mechanisms of action, as well as tolerable toxicity [15,16].

Coconut palm (*Cocos nucifera*, *Arecaceae*) is native to Southeast Asia and India, and is considered one of the essential fruit palms in the world [17]. Besides edible applications due to high nutritional value, they can produce biodegradable detergents, cleaning products, cosmetics, toiletries, and plasticizers [18]. The coconut fruit ethanolic extract reveals the presence of phenols, tannins, anthocyanidins, flavonoids, triterpenes, steroids, and alkaloids, while the oil has been reported to contain medium-chain fatty acids. The edible oil attains antioxidant, anti-inflammatory, antiviral, anti-inflammatory, antimicrobial, and immunomodulatory efficacies and has been reported to have emollient as well as wound healing efficacies [3].

**Figure 1:**
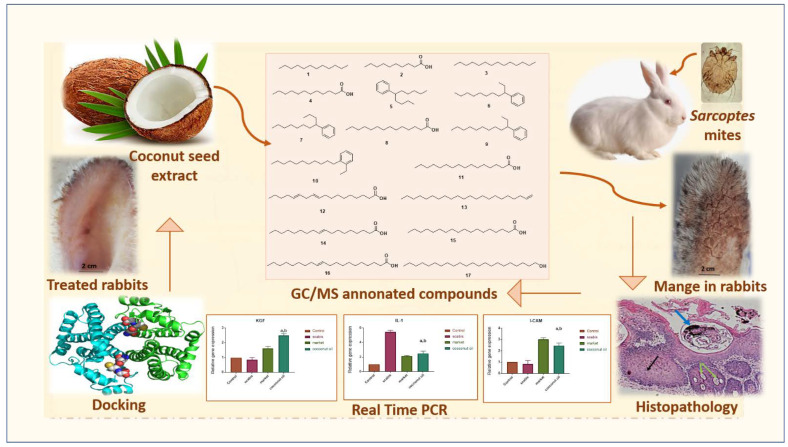
The general summary of the present research is as follows: the phytochemical composition as well as GC-MS profiling of coconut seed extract (CSE) was employed. Additionally, the scabicidal potential of CSE on sarcoptic mange in rabbits was investigated via in vitro, in vivo, histopathology, mRNA expression, and docking analysis.

In the present study, the phytochemical composition as well as GC-MS profiling of coconut seed extract (CSE) was employed. Additionally, the scabicidal potential of CSE on sarcoptic mange in rabbits has been investigated via in vitro, in vivo, histopathology, mRNA expression, and docking analysis for the first time to open the gate for incorporating natural candidates towards proper and safe management of sarcoptic mange in rabbits. Figure 1 depicts the framework of the present investigation.

## 2. Results

### 2.1. GC-MS Profiling of Coconut Seed Extract

**Figure 2:**
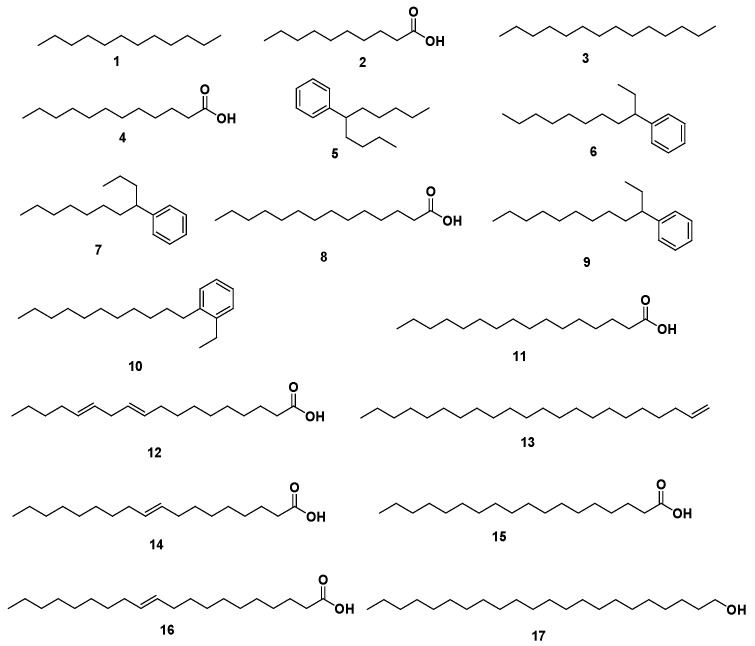
Structures of identified compounds from coconut seed oil by GC/MS analysis.

A total of 17 compounds (Figure S1, Table 1, and Figure 2) belonging to different phytochemical classes were identified representing 97.69% of the total detected compounds including fatty acids (58.57%), phenyl-alkane (37.56%), and acyclic alkanes derivatives (1.56%) (Table 1). A total of 8 fatty acids (58.57%), representing the major identified class were identified, varying from 5 saturated fatty acids (38.85%) to 2 mono-unsaturated fatty acids (19.54%), and 1 polyunsaturated fatty acid (0.18%). The total SFA content was greater than that of the total unsaturated fatty acid (UFA) and accounted for 12.22% lauric acid **4**, 9.24% myristic acid **8**, 8.01% palmitic acid **11**, and 6.82% stearic acid **15**. Additionally, 5 phenyl-alkane compounds (37.56%) were identified.

### 2.2. Phytochemical Investigation of Coconut Seed Extract

**Figure 3:**
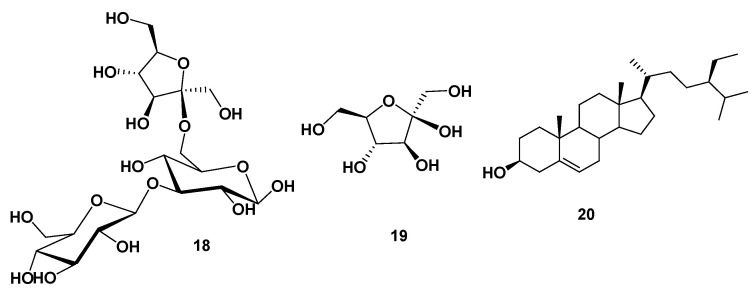
Structures of isolated compounds from coconut seed extract.

Based on the physicochemical and chromatographic properties, the spectral analyses from UV, 1H, and DEPT-Q NMR, as well as comparisons with the literature, the seed extract of *C. nucifera* seeds afforded the following known compounds: 3″(1‴-*O*-*β*-D-glucopyranosyl)-sucrose **18** [19], *β*-D-fructofuranoside **19** [19], and *β*-sitosterol **20** [20] (Figure 3, NMR spectra are displayed in Figures S1–S7 in the Supplementary Materials).

### 2.3. The Antioxidant Potential of Coconut Seed Extract

**Figure 4:**
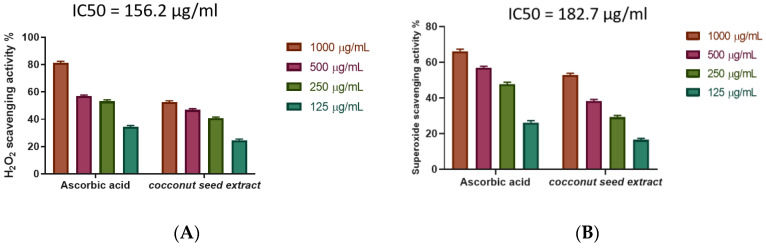
(**A**) Coconut seed extract potential of H_2_O_2_ scavenging at different concentrations (1000 µg/mL, 500 µg/mL, 250 µg/mL, and 125 µg/mL). Bars represent mean ± standard deviation (SD). (**B**) Superoxide radical scavenging activity of coconut seed extract at different concentrations (1000–125 µg/mL). Bars represent mean ± SD. Significant difference between groups is analyzed by a two-way ANOVA test.

The study investigated the antioxidant activity of CSE as a scavenger potential against (H_2_O_2_). In line with the findings, the utmost H_2_O_2_ radical scavenging activity of CSE was 55.55% at a 1000 µg/mL concentration. In comparison with ascorbic acid as a standard (IC50 = 156.2 µg/mL), CSE suppressed the formation of H_2_O_2_ in an exceedingly dose-dependent manner (Figure 4A, Table S3).

The superoxide scavenging (SOD) activity of CSE was resolute. The scavenging potential of CSE and the standard increase are concentration dependent (Figure 4B, Table S4), with CSE showing the most superoxide radical scavenging activity of 52.7% at a 1000 µg/mL concentration, IC50 147.1 µg/mL.

### 2.4. Evaluation of the In Vitro Scabicidal Potential of Coconut Seed Extract

The coconut seed extract of 20% had a remarkable acaricidal effect according to in vitro results, where the mites experienced a sluggish movement which started at 1 h post application (PA) and ended with 99% mortality at 24 PA. This was confirmed by microscopic examination.

### 2.5. Evaluation of the In Vivo Efficacy of Coconut Seed Extract on Infected Rabbits

**Figure 5:**
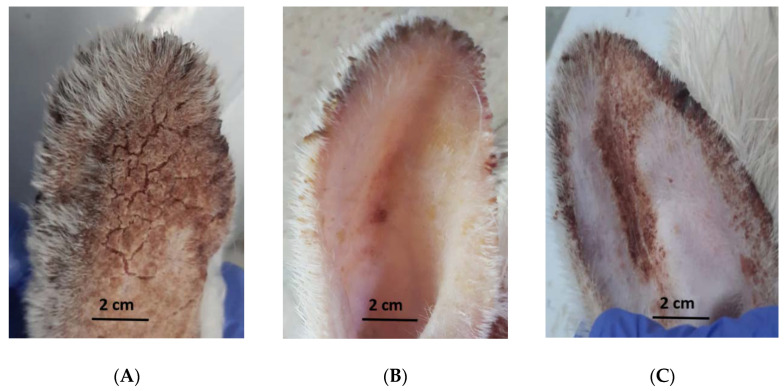
Macroscopical examination of infected rabbits with mange at the end of the experiment. (**A**) control group; (**B**) coconut seed extract group; and (**C**) ivermectin group.

Rabbits infected with *Sarcoptes scabiei* showed sarcoptic mange on and inside the ears with some chronic lesions and scabs with asbestos appearance. These animals were in a bad condition suffering from itching, congestion, and scratching in the infected ears which were directed downwards, as well as anorexia. The rabbits treated with CSE exhibited improvement of clinical signs which started gradually from day 4 PA until the end of the experiment (3 weeks PA). The recovery was expressed through observation of the absence of itching, bleeding, scale formation, and restlessness, while signs of improvement appeared as smoother skin and new hair growth [7]. On the other hand, the ivermectin-treated animals showed gradual improvement of the signs of mange, starting from day 7 PA until the end of the experiment but without complete eradication (Figure 5).

On day 5 PA, the skin scrapings from infected animals in both groups—CSE and ivermectin—were found to contain dead mites, which completely disappeared by the next examination time at day 10.

### 2.6. Histopathological Investigation

**Figure 6:**
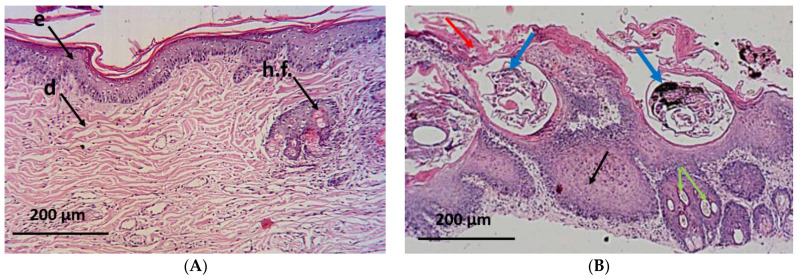

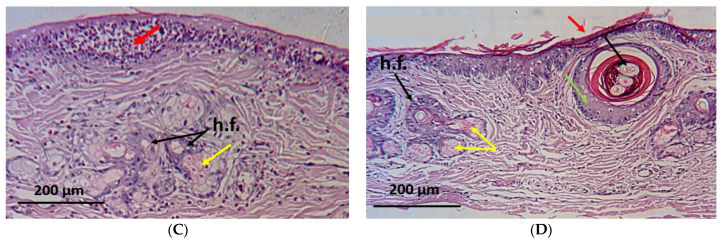
Microscopical examination of skin from different groups of animals. (**A**) normal group showing normal architecture of the skin: e—epidermis, d—dermis, and h.f.—hair follicles; (**B**) positive control group showing skin damage with hyperkeratosis (red arrows), mites at different stages embedded in the skin (blue arrows), hypergranulation of the dermis (green arrows), and severe akanthosis with cellular infiltration (black arrows); and (**C**) coconut seed extract group showing restoration of normal architecture, with only mild cellular infiltration (red arrow). Healthy sebaceous glands (yellow arrow) and hair follicles (black arrows); (**D**) ivermectin group showing moderate damage with hyperkeratosis (red arrow), mature mites with eggs still embedded in the dermis (black arrow) surrounded by cellular infiltration (green arrow), and some sebaceous adenitis (yellow arrows).

The histopathological examinations of the normal group showed the appearance of the normal architecture of the skin consisting of the epidermis and the dermis. The epidermis consisted of stratum corneum (layers of dead, scaly, and keratinized cells) and stratum granulosum (layers of flattened keratinocytes). The dermis shows the reticule layer and hair follicles as well as sebaceous and sweat glands (Figure 6A). On the other hand, skin specimens from the positive control group showed an altered histology which is typical for this parasitic infection [21]. The skin appeared damaged and folded with a wavy epidermis, hyperkeratosis, akanthosis and sloughing of the stratified squamous epithelial, leading to skin erosion. Moreover, necrotic debris mixed with different developmental stages of the mites could be seen within the epidermal burrows surrounded by inflammatory cellular infiltration as well as hypergranulation areas of the dermis (Figure 6B).

Interestingly, biopsy specimens from the CSE-treated animals showed improvement in the dermis and the epidermis with a mild cellular infiltration, absence of acanthosis or mites, proliferation of the hair follicles, and appearance of healthy sebaceous glands (Figure 6C). Meanwhile, a moderate progress in the skin state was shown in the ivermectin-treated group where some inflammatory cells were observed at the end of the study along with moderate hyperkeratosis. A few mature mites (with eggs) appeared embedded in the dermis, surrounded by areas of cellular infiltration, with the presence of some areas of sebaceous adenitis (Figure 6D).

### 2.7. Gene Expression Results

**Figure 7:**
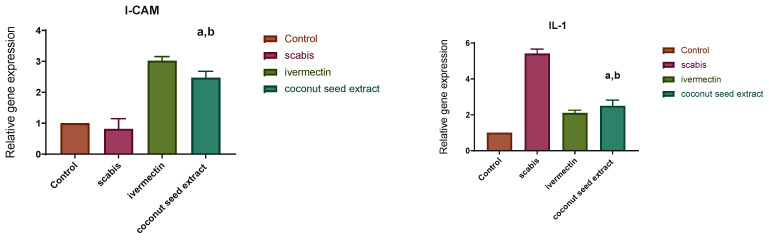

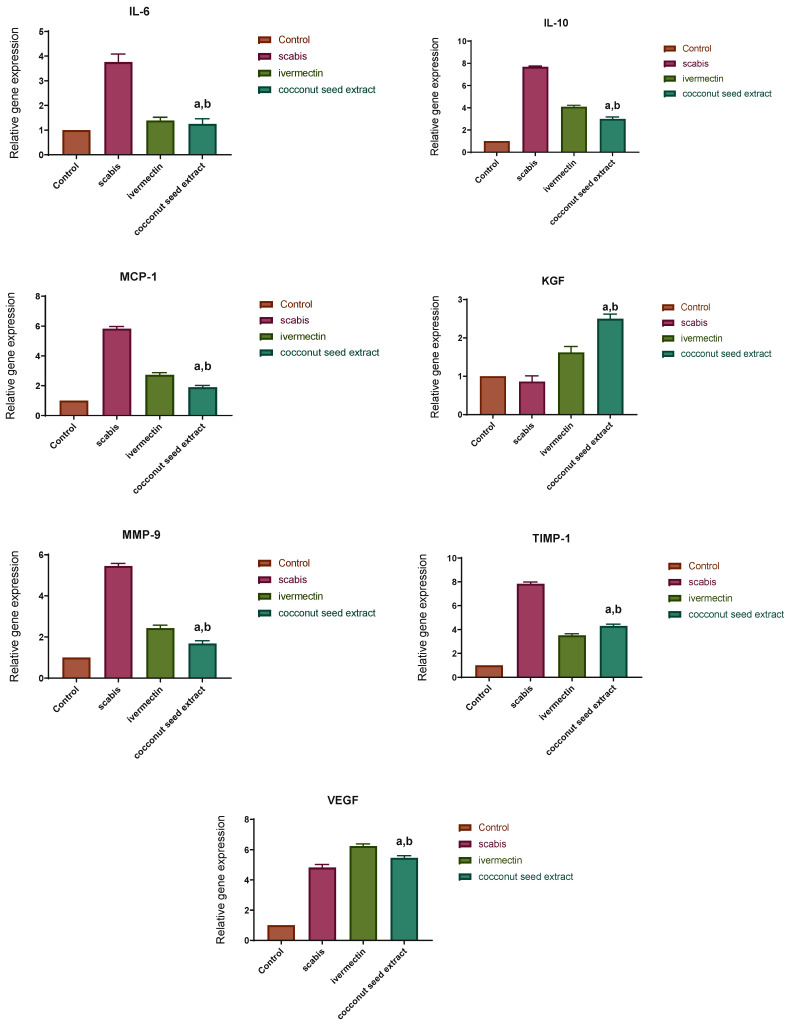
Relative gene expression in skin tissue of different animal groups using qRT-PCR. After normalization to GAPDH, the data reflect a fold in expression compared with the normal control group. The bars represent mean ± SD. To determine if there is a significant difference between groups, a one-way ANOVA test was performed with (a) *p* < 0.05 compared with the normal control group and (b) *p* < 0.05 compared with the market-drug-induced group.

The results of q-PCR revealed that animals treated with CSE exhibited a downregulation of the pro-inflammatory cytokines (IL-1β, IL-6) as well as the pleiotropic cytokine (IL-10) and the monocyte chemoattractant protein-1 (MCP-1). On the other hand, VEGF, ICAM-1, KGF, MMP-9, and TIMP-1 were upregulated two- to seven-fold (Figure 7).

### 2.8. Molecular Docking Study

In attempt to investigate the mechanistic scabicidal potential of CSE, an in silico study was conducted on the 20 identified compounds via screening against 3 different crucial protein targets which are extensively incorporated in the process of infection. The selected targets include IL-1β which play an effective role in the stimulation of regulatory T cells and IL-6 which is incorporated in Th17 cell generation and IL-17 secretion, in addition to VEGF as a promotor of blood supply formation. Significantly, the GSH enzyme is associated with the resistance mechanism of *Sarcoptes* mites towards cidal agents [22]. The computational program (MOE 2019.010) for the ligand’s validation and visualization of the different docked poses was employed. The protein IL-1β is represented by protein (PDB ID code: 6Y8M), co-crystallized with its inhibitory ligand SX2 (a bromo amido pyridine derivative); IL-6 is represented by (PDB ID code: 1ALU), co-crystallized with its ligand (tartaric acid); VEGF is represented by protein (PDB ID code: 4AG8), co-crystallized with its new inhibitory ligand “axitinib”; and that of the mite delta class GST is represented by protein (PDB ID code: 3EIN).

**Figure 8:**
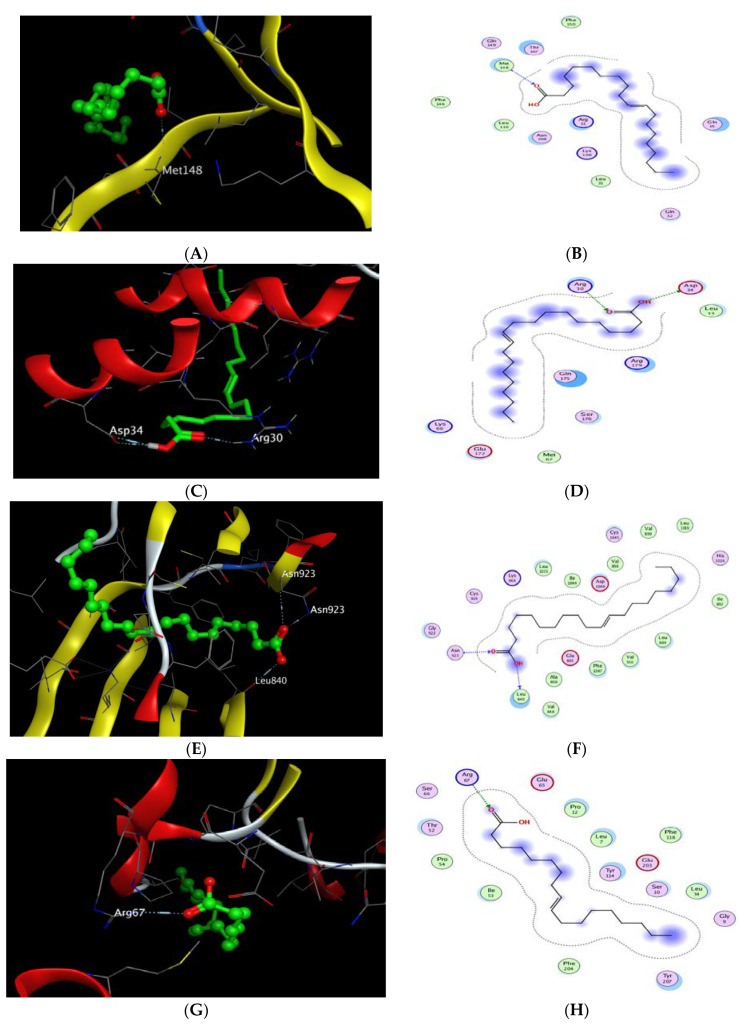

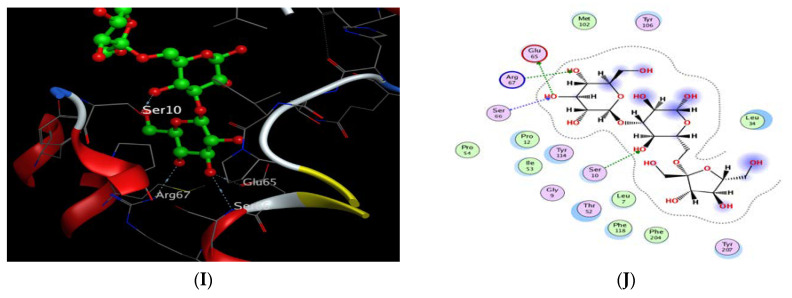
(**A**) 2D interactions and (**B**) 3D docking pose of gondoic acid in the active pocket site of IL-1β (PDB: 6Y8M); (**C**) 2D interactions and (**D**) 3D docking pose of gondoic acid in the active pocket site of IL-6 (PDB: 1ALU); (**E**) 2D interactions and (**F**) 3D docking pose of gondoic acid in the active pocket site of VEGF (PDB: 4AG8); (**G**) 2D interactions and (**H**) 3D docking pose of oleic acid in the active pocket site of GST (PDB: 3EIN); (**I**) 2D interactions and (**J**) 3D docking pose of 3″(1‴-*O*-*β*-D-glucopyranosyl)-sucrose in the active pocket site of GST (PDB: 3EIN).

The X-ray crystallographic structure of (IL-1β) complexed with its ligand was obtained from the Protein Data Bank through the internet (http://www.rcsb.org/pdb/, code 6Y8M) accessed on 2 June 2022. For validation, the ligand was re-docked with the active pocket with a good RMSD value of 1.311 and energy score of −5.870 kcal/mol. The results showed 5 hydrogen bond interactions with Met 148, Thr 147, Gln 149, and Arg 11 as H-acceptors and with Met 148 as the H-donor, as well as 1 ionic interaction with Arg 11. The docking scores of the 20 compounds detected in CSE against IL-1β (6Y8M) are summarized in Table S5. Docking results revealed that gondoic acid achieved a docking score of −5.817 kcal/mol, which was relatively lower than that of the co-crystallized ligand with one hydrogen bond interaction as the H-acceptor with Met 148, resembling that of the co-crystalized ligand (Table 2, Figure 8A,B). It is worth mentioning that most of the other investigated compounds achieved approximately the same co-crystalized ligand energy score ranging from −5.042 to −5.362 kcal/mol.

Regarding docking with IL-6, its X-ray crystallographic structure complexed with its ligand (tartaric acid) was obtained from the Protein Data Bank through the internet (http://www.rcsb.org/pdb/, code 1ALU) accessed on 2 June 2022. The docking algorithm was able to predict the co-crystalized ligand pose with RMSD 1.758 and an energy score of −4.191 kcal/mol (Table 3). The pattern of interactions was distributed through the H-acceptor with amino acid residues Arg 182 and Arg 179 in addition to ionic interactions with the same residues besides one hydrogen bond with Gln as the H-donor. Interestingly, the docking results of the 20 compounds revealed their better affinities towards IL-6 than the co-crystalized ligand (Table S6). Gondoic acid and 1-docosanol achieved the lowest binding energies with ΔG −5.291 and −5.308 kcal/mol, respectively. However, 1-docosanol showed higher RMSD than gondoic acid, where it showed 2 H-bond interactions through the carboxylic hydroxyl group with Asp 34 as the H-donor and one H-bond acceptor with Arg 30 through the carbonyl group (Figure 8C,D).

Moreover, docking with VEGF (PDB ID code: 4AG8) was represented by co-crystallization with its inhibitory ligand, axitinib, where the X-ray crystallographic structure was obtained from the Protein Data Bank. Upon validation of the docking, the ligand possessed an energy score of −7.970 kcal/mol with RMSD 0.9175, attaining 2 H-bond interactions at the binding site with the amino acid residues Asn 923 and Cys 919 as the H-bond acceptor and two pi-H interactions with Leu 840. The docking results of the 20 compounds are listed in Table S7, where gondoic acid showed a higher affinity to VEGF with an energy score of −8.362 kcal/mol, which was lower than that of the ligand with a score of −7.970 kcal/mol (Table 4). Moreover, they showed the same interacting pattern with 2 H-bond interactions as the H-acceptor with Asn 923 via its carbonyl group and 1 H-bond with Leu 840 as the H-donor (Figure 8E,F). Remarkably, compounds 10,13-octadecadienoic acid, 1-docosene, and 1-docosanol achieved energy scores lower than those of the co-crystallized ligand of −8.180, −8.111, and −8.178, respectively.

Finally, the X-ray crystallographic structure of the delta class GST of *Drosophila melanogaster* was obtained from the Protein Data Bank via (http://www.rcsb.org/pdb/, code 3EIN) accessed on 2 June 2022. The redocking of the co-crystalized ligand, glutathione, showed 4 H-bond interactions: 2 interactions with Arg 67 and Ser 66 as the H-acceptor and the other 2 interactions with Glu 65 and Ile 53 as the H-donor, in addition to 2 ionic interactions with Arg 67 and Glu 65, with an energy score of −5.945 kcal/mol (Table 5). The docking results of the 20 compounds enlisted in Table S8 showed superiority for palmitic acid, 10,13-octadecadienoic acid, 1-docosene, oleic acid, 1-docosanol, 3″(1‴-*O*-*β*-D-glucopyranosyl)-sucrose, and *β*-sitosterol in their affinities towards glutathione with ΔG ranging from −6.010 to −7.240 kcal/mol. It was found that oleic acid exhibited 1 H-bond interaction with amino acid residue Arg 67 as the H-acceptor (Table 5, Figure 8G,H), while 3″(1‴-*O*-*β*-D-glucopyranosyl)-sucrose achieved 4 H-bond interactions, of which 3 interactions were as the H-acceptor with Ser 10, Arg 67, and Ser66 and 1 interaction with Glu 65 as the H-donor in a similar pattern to the co-crystalized ligand (Table 5, Figure 8I,J).

## 3. Discussion

The pathogenesis of scabies is quite complicated and various mechanisms are implicated such as the persistence of the parasite, microvascular/endothelial dysfunction, aggravated immune response (via the imbalance between pro-inflammatory and anti-inflammatory cytokines), as well as the induction of permanent oxidative stress caused by the mites burrowing deep into the skin, which directly affects both structure and function of the skin [23]. All these factors combine to hinder proper treatment, especially with the fact that most synthetic agents act on mite killing rather than immunomodulation or tissue healing [4]. Accordingly, plant-derived phytochemicals with wide therapeutic potential with negligible side effects can safely act as alternatives for synthetic candidates for the eradication of infectious diseases [16,24]. In this sense, the coconut palm has been reported to possess antioxidant, anti-inflammatory, antimicrobial, and immunomodulatory properties with an emphasis on the presence of a high content of lauric, palmitic, stearic, and oleic acids which have proved efficacy against scabies in a number of studies [25,26,27,28]. It showed antibacterial activity against *Staphylococcus aureus* and *Candida* skin infections such as oral and vaginal candidiasis with significant results [29]. Interestingly, studies have demonstrated that coconut seed oil may alter gene expression in inflammatory responses, with suppression of pro-inflammatory cytokines and enhancement of protective barriers in the skin [30]. To the best of our knowledge, the acaricidal effect of CSE against *Sarcoptes scabeiei* has never been investigated.

Hence, the present study investigated the GC/MS composition as well as the phytochemical composition of crude seed extract and evaluated the scabicidal potential of the CSE against scabies mites in both in vitro and in vivo assays. The GC-MS analysis revealed the presence of a prevailing percentage of oleic, palmitic, and stearic acids, known as medium-chain fatty acids, which is a further encouraging step as they are reported to demonstrate repellent activities against many arthropods [31]. The application of CSE did not reveal any signs of skin irritation, inflammation, or restlessness during and after the application, coincident with the reported safety of topical coconut extract [32]. The safety of the oil was ascertained by studies that reported the efficacy of coconut palm as antifungal, antibacterial, anti-inflammatory, and anti-eczema without allergic outputs [18]. Our results revealed that CSE could exert a cidal effect on *Sarcoptes scabiei* mites 24 h post application, which shows a strong acaricidal effect, as reported [6,31]. The mites’ death positively affected the inflammatory reactions in the animals’ skin, which started to show healthy signs such as stoppage of inflammation and hyperkeratosis, appearance of new skin layers, and growth of new hair, which was coincident with reports of successful treatment of rabbit mange [14]. This complete recovery was observed after 3 weeks, while the healing of the ivermectin group extended to the end of the experiment (4 weeks) without complete healing. The histopathological results showed improvement in both the dermis and the epidermis, expressed in a decrease in inflammatory cells as well as an absence of remnants of mites in the skin layers of the treated animals [6]. The improvement is mainly due to the death of mites and cessation of inflammation, pruritis, skin destruction, and scale formation [21]. On the other hand, slow changes occurred to the skin layers in the ivermectin group during treatment, where some inflammatory cells as well as remnants of dead mites were still observed at the end of the study. This may be attributed to the concentrated effects of ivermectin towards mites, as well as the normal itching and allergic sensations caused by ivermectin topical application, which prolongs inflammation and further delays the appearance of healthy signs [33].

To gain insight into the modulative effects of CSE on the pathophysiology of scabies, epidermal keratinocytes as the first line of defense against harmful external invaders should be implemented [34]. Keratinocytes attain recognition receptors on their surfaces, allowing them to recognize various pathogens and initiate immune responses, via secretion of cytokines, chemokines, and anti-microbial peptides, which contribute to the recruitment of inflammatory cells [34,35]. Keratinocytes play a significant role in the regulation of skin immune homeostasis and any imbalance in their activities can develop a disease [36,37,38]. Our results showed that a large number of genes in both skin keratinocytes and fibroblasts are differentially expressed in response to live burrowing scabies mites, or their products (as saliva or eggs), which further activate other cell types [39,40]. Thus, there is a complex interplay (cross talk) among many other cell types in the skin (including lymphocytes, endothelial cells, LCs, and dendritic cells) in response to scabies, leading to evolution of inflammation and oxidative stress states [40]. This might enhance reactive oxygen species such as hydrogen peroxide (H_2_O_2_), which induces lipid peroxidation and causes harmful alterations to both structure and permeability of the skin [41,42,43]. In our study, amelioration of the altered oxidant/antioxidant balance towards normal in treated animals indicated the potential antioxidant action of coconut, supported by faster clinical and parasitological recovery. Antioxidants are thought to help manage wound oxidative stress and hence speed up wound healing [44]. They play a critical role in controlling the damage that biological components such as DNA, protein, lipids, and body tissue may sustain in the presence of reactive species [44]. The ROS scavenging potential of the extract might be the cornerstone in the management protocol since high levels of reactive oxygen species (ROS) at the wound site are the main promotor of collagen breakdown, destruction of the extracellular matrix (ECM), reduction in angiogenesis and re-epithelialization, and increasing pro-inflammatory cytokines, all which prolong inflammation [44,45].

Reports state that penetrating mites sensitize the skin keratinocytes and tend to suppress the body’s immunity by downregulating the gene expression of i-CAM-1 (an intracellular adhesion molecule on the endothelial cell surface), which reduces the supply of blood and immunity cells to the area of penetration and diminishes both lymphocytes’ and neutrophils’ protective mechanisms [46]. On the contrary, the infection upregulates the chemokine MCP-1, which activates immune cells to promote inflammation [47]. Likewise, the regulatory T cells (type 1) are elicited to secret IL-10, which is an anti-inflammatory cytokine that plays a crucial role in preventing inflammatory and autoimmune pathologies in humans so clinical symptoms are unobserved until 4–6 wks after a person becomes infested with scabies mites [11,46]. Additionally, the keratinocytes sensitized by mite products tend to increase expression of VEGF, which is also increased by the mites themselves to increase angiogenesis, through which the mites enhance the surrounding blood supply to obtain requirements from the food, a process that further exaggerates inflammation [11]. As a result, KGF receptor signaling is diminished, which further reduces the proliferation rate of epidermal keratinocytes at the wound edge, resulting in delayed re-epithelialization of the wound. The inflammation also causes a significant elevation of matrix metalloproteinase (MMP-9) which belongs to MMPs—a group of hydrolase enzymes expressed in many overwhelming cases such as wounds, osteoarthritis, ischemia, and infectious diseases [46,48]. MMP-9 is implemented in nearly all parasitic infections for purposes of tissue remodeling and generally results in the downregulation of ECM syntheses such as collagen II and aggrecan [49]. The biological activities of MMPs are strictly controlled by TIMP-1 (tissue inhibitor of metalloproteinase) and upon the imbalance in the MMPs/TIMPs ratio, proteolysis occurs [50].

**Figure 9:**
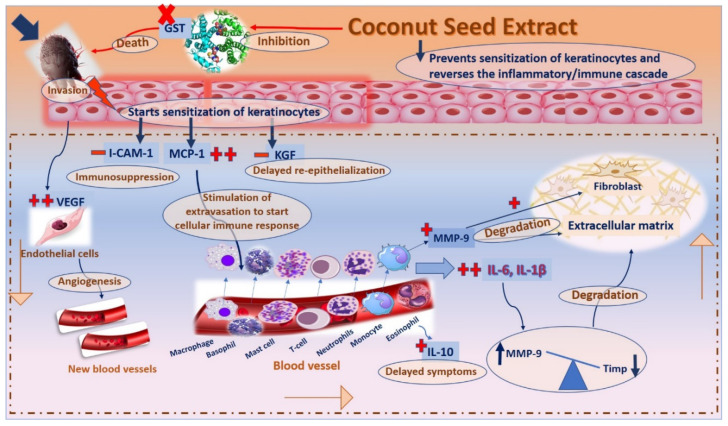
A schematic diagram for the proposed mechanism of action of coconut seed extract on scabies-infected rabbits.

Accordingly, reversing the activity of this series of interconnected genes may act as a valuable therapeutic strategy in scabies progression [51]. The topical application of CSE led to a significant decrease in the expression of IL-1β, IL-6, IL-10, MMP-9, VEGF, and MCP-1, and an increased gene expression level of i-CAM-1, KGF as well as TIMP-1, all of which are expressed in Figure 9 [51]. As a result, this led to a decrease in pro-inflammatory cytokines and an increase anti-inflammatory ones as well as an improvement in host immunity towards invading mites, which could reverse the unhealthy signs and result in decreasing inflammation, improving re-epithelialization and fast recovery [50,51].

The in silico docking was performed on the individual phytoconstituents of CSE to present a computational explanation to the previous findings and also explain whether the extract could affect the life cycle of the mites themselves causing their death [52]. The results clearly showed that most compounds acquired sufficient affinity towards the screened proteins, especially gondoic acid (11-eicosenoic acid) which possessed higher affinities towards IL-1β, IL-6, and VEGF with higher binding energy scores [53]. Interestingly, among many compounds which showed good affinities towards the GST protein, the sugar compound 3″(1‴-*O*-*β*-D-glucopyranosyl)-sucrose showed the highest affinity score. GST is a chief enzyme for mites which might contribute to insecticidal resistance by playing a central role in the detoxification of xenobiotic and endogenous compounds in mites [22]. It should be taken into consideration that studies reported the acaricidal effect of sugar derivatives such as erythritol, which was reported as a safe insecticide [52].

Collectively, it is clear that the fatty acids content of coconut seed extract may share a great role in the fast recovery of animals from scabies, which explains why coconut seed extract was used in a previous study side by side with oils as a cream base for aloe vera gel to treat skin damages occurred with scabies infection [54,55]. Previous studies reported the anti-inflammatory, skin permeation, and enhancement of the angiogenesis of FAs, where they moisturize the dry desquamated skin by traversing deeper into the different layers of the skin, diminish the fibroblasts and other inflammatory cells in the scarring tissues, and promote collagen formation which enhances wound healing [56,57]. This was coincident with a previous study which stated that FAs contained in coconut seed have soothing and curative effects on the skin, as well as hair-growth-promoting effects which greatly aid in clinical recovery via the enhancement of skin smoothness and fur growth in rabbits [58].

## 4. Material and Methods

### 4.1. Collection of Plant Material

*C. nucifera* seeds were collected on March–April 2021 from fresh fruits in India and were kindly identified by Dr. Abd El-Halim A. Mohammed of the Horticultural Research Institute, Department of Flora and Phytotaxonomy Research, Dokki, Cairo, Egypt. A voucher specimen (2021-BuPD 81) was deposited at the Department of Pharmacognosy, Faculty of Pharmacy, Beni-Suef University, Egypt.

### 4.2. GC/MS Analysis, Extraction, Fractionation, Isolation of Phytoconstituents, and In Vitro Antioxidant Potential of Coconut Seed Extract

Plant material, chemicals, reagents, spectral analyses, extraction [20,44,59,60,61,62,63], fractionation [64,65,66,67,68,69,70], and GC/MS analysis [62,71,72,73,74] of *Cocos nutifera* seeds are discussed in detail in the Supplementary Materials.

### 4.3. Biological Investigation

#### 4.3.1. Collection of *Sarcoptes scabiei* Mites

The adult mites were collected from naturally infected rabbits at an animal house in Deraya University, Minia, Egypt. A total of 4 groups of 4 infected rabbits each were employed in the experiment in compliance with the guidelines for the care and use of laboratory animals of the National Institute of Health, under the approval number of 1/2022 of the ethical committee at Deraya University. Free access to food and water was provided, where all rabbits were fed using steel hoppers and provided water ad libitum.

The infected skin samples were scraped from the edges of the lesions into aseptic plastic tubes, transferred to Petri dishes, and incubated at 35 °C for 30 min in a biochemical oxygen demand incubator (BOD).

#### 4.3.2. In Vitro Application of Coconut Extract on Sarcoptic Mange

Mites were placed in a Petri dish containing 2 mL of diluted extract (20%) and then the dishes were incubated in BOD. Observations of the response were recorded at 1 h, 12 h, and 24 h post application. Petri dishes, including a negative control group (with only distilled water) and a positive one with 5% ivermectin (1 cm^3^/L), were incubated at a relative humidity of 75% and a temperature of 25 °C. The mite death was confirmed by needle stimulation of mites which were considered dead if they gave no reaction upon stimulation.

#### 4.3.3. In Vivo Application of Coconut Seed Extract

The experiment was conducted for 4 weeks on adult male rabbits (2.8–3.2 kg) exhibiting clinical signs of mange infection in their ears with hyperkeratinization, inflammation, redness, itching, and irritability, which was further confirmed by microscopic detection of mites in skin scrapings. A total of 20 rabbits were divided into 4 groups of 5 as follows: normal group, positive control group (paraffin oil), ivermectin-treated group (5% ivermectin), and the CSE group (20% CSE in paraffin oil). Paraffin oil (mineral oil) was chosen as a diluent for the CSE group as it has negligible effects on mites as reported in [75]. Each group was treated via dipping the infected ears once daily, with each rabbit housed in a separate cage. For the evaluation of clinical recovery, the rabbits were examined every 2 days to detect onset of clinical recovery of symptoms (itching, scratching, and anorexia) and improvement of lesions (cessation of scab formation, smoothing of skin, absence of inflammation and redness, and starting of hair growth) [76]. Every three days, skin scrapings were collected from infected and recovered areas of each rabbit and were microscopically examined during the course of treatment to detect sarcoptic mites [76].

#### 4.3.4. Histopathological Examination

Tissue samples were obtained from normal and infected ears at 0 and 3 weeks from the start of treatment with 20% CSE and ivermectin. Next, samples were fixed in 10% buffered formalin, dehydrated in ascending grades of ethyl alcohol, cleared with xylene, impregnated in melted paraffin at 55–60 °C, and finally, embedded in paraffin wax. Paraffin-embedded tissue sections “3–5 µm thick” were deparaffinized, rehydrated, and subjected to hematoxylin and eosin (H & E) stain and investigated with a light electron microscope [77].

#### 4.3.5. RNA Isolation and qRT-PCR Assay

An amount of 100 mg of the infected tissue was homogenized using a digital homogenizer (Branson Digital homogenizer^®^, Danbury, CT, USA) in 1 mL of TRIzol™ RNA Extraction Reagent (Amresco, Solon, OH, USA). The biopsy specimen RNA extraction was performed according to the manufacturer’s protocol. The extracted RNA was used to synthesize cDNA for comparable quantities of the total RNA in all samples following the manufacturer’s protocol using the RevertAid H Minus First Strand cDNA Synthesis Kits (#K1632, Thermo Science Fermentas, St. Leon-Ro, Germany). Using the cDNA as a template, the qRT-PCR was performed on the Applied Biosystems Step One Plus. NCBI primer blast software was used to design the primers which were manufactured by Invitrogen. Data were analyzed according to the 2^−ΔΔct^ method using the GAPDH gene as a housekeeping gene [44]. Sequences of the used primers are listed in Table S1, and gene expression values are listed in Table S2 in the Supplementary Materials.

### 4.4. Molecular Docking Study

The X-ray crystallographic structures of protein targets (of the catalytic domain) in complexation with erlotinib were obtained from the Protein Data Bank via the internet (http://www.rcsb.org/pdb, accessed on 2 June 2022). All molecular modeling calculations and docking studies were conducted using the Molecular Operating Environment 2019.0102 software (MOE) [78]. The preparation of the protein included the removal of water molecules and uninvolved ligands by using the quick preparation tool in MOE, applying the default options. Docking of the conformation database of the target compounds was carried out after preparation of the enzyme. The following methodology was generally applied: the enzyme active site was located by the site finder tool and the docking tool was initiated. The program specifications were adjusted to ligand atoms as the docking site and alpha triangle as the placement methodology to be used. The scoring methodology London dG was used and was adjusted to its default values. The MDB file of the ligand to be docked was loaded and dock calculations were run automatically. Receptor-ligand interactions of the complexes were examined in 2D and 3D styles and the poses that showed the best ligand–enzyme interactions were selected and stored for energy calculations. The selection of poses was conducted according to the better obtained binding scores and RMSD Refine values; the obtained scores, RMSD Refine values, and interactions with the binding pocket site of the enzymes are discussed in [79].

### 4.5. Statistical Analysis

Graph Pad Prism version 9 statistical software was used to tabulate the data (GraphPad, La Jolla, CA, USA). The one-way analysis of variance (ANOVA) test followed by the Bonferroni post hoc test for multiple comparisons were used to assess statistical differences between the groups. Statistical significance is defined as a *p*-value of less than or equal to 0.05.

## 5. Conclusions

Effective control of scabies based on the current acaricidal market agents has proved to be a huge challenge, with reports of emerging resistance as well as treatment failure. This study proved the acaricidal efficacy of coconut seed extract against mange mites in rabbits with a comparable cidal efficacy but with better tissue healing effects than some conventional synthetic agents, such as ivermectin, which is the gold standard of the study. The coconut seed extract proved efficacy in healing the harmful effects of mange on rabbits, expressed in morphological, histopathological, and gene analysis studies. The coconut seed extract offers an effective, low-cost, safe, and ecofriendly candidate that can be an excellent alternative to commercial drugs used for the control of arthropods with harmful impacts on human and animal health. Such candidates can be effectively used to build novel biocides for use in crop advancement as well as livestock protection.

## Figures and Tables

**Table 1:** *Cocos nucifera* seed oil composition using GC/MS analysis.

No.	Identified Compound	MF	Area %	RT	RI
**1**	Dodecane	C_12_H_26_	0.09	9.77	928
**2**	Capric acid	C_10_H_20_O_2_	2.56	12.79	903
**3**	Tetradecane	C_14_H_30_	0.54	14.18	973
**4**	Lauric acid	C_12_H_24_O_2_	12.22	17.04	911
**5**	Benzene, (1-butylhexyl)-	C_16_H_26_	3.47	18.13	920
**6**	Benzene, (1-ethylnonyl)-	C_17_H_28_	6.57	18.62	910
**7**	Benzene, (1-propyloctyl)-	C_17_H_28_	6.96	19.83	974
**8**	Myristic acid	C_14_H_28_O_2_	9.24	21.51	901
**9**	Benzene, (1-ethyldecyl)-	C_18_H_30_	14.39	22.06	932
**10**	Benzene, (1-ethylundecyl)-	C_19_H_32_	6.17	23.71	904
**11**	Palmitic acid	C_16_H_32_O_2_	8.01	24.73	900
**12**	10,13-Octadecadienoic acid	C_18_H_32_O_2_	0.18	24.88	901
**13**	1-Docosene	C_22_H_44_	0.79	25.35	956
**14**	Oleic acid	C_18_H_34_O_2_	19.09 *	27.45	939
**15**	Stearic acid	C_18_H_36_O_2_	6.82	27.74	921
**16**	Gondoic acid	C_20_H_38_O_2_	0.45	29.90	929
**17**	1-Docosanol	C_22_H_46_O	0.14	31.08	905
Total			97.69%		

RI: retention index relative to *n*-alkanes, RT: retention time (min), MF: molecular formula, *: major compound, %: percentage.

**Table 2:** Receptor interactions and binding energies of gondoic acid and ligand into the active pocket site of IL-1β catalytic domain.

Cpd.	S ^a^ kcal/mole	RMSD_Refine ^b^	Amino Acid Bond	Distance Å	E (kcal/mol)
Gondoic acid	−5.817	1.684	MET 148/H-acceptor	3.19	−1.00
Ligand	−5.87	1.311	MET 148/H-donor	3.04	−2.90
			MET 148/H-acceptor	3.08	−0.80
			THR 147/H-acceptor	2.74	−5.00
			GLN 149/H-acceptor	2.98	−3.50
			ARG 11/H-acceptor	3.15	−5.00
			Arg 11/Ionic	2.98	−4.60

^a^ S: The score of a compound placement inside the protein binding pocket. ^b^ RMSD_Refine: the root-mean-square deviation (RMSD) between the predicted pose and those of the crystal one (after and before the refinement process, respectively).

**Table 3:** Receptor interactions and binding energies of gondoic acid and ligand into the active pocket site of IL-6 catalytic domain.

Cpd.	S ^a^ kcal/mole	RMSD_Refine ^b^	Amino Acid Bond	Distance Å	E (kcal/mol)
Gondoic acid	−5.291	1.31	Asp 34/H-donor	3.22	−3.70
			Asp 34/H-donor	3.18	−0.60
			Arg 30/H-acceptor	3.05	−1.50
Ligand	−4.191	1.758	Gln 175/H-donor	2.78	−1.60
			Arg 182/H-acceptor	2.49	−3.50
			Arg 182/Ionic	3.06	−4.10
			Arg 179/H-acceptor	2.61	−4.30
			Arg 179/Ionic	2.51	−8.70

^a^ S: The score of a compound placement inside the protein binding pocket. ^b^ RMSD_Refine: the root-mean-square deviation (RMSD) between the predicted pose and those of the crystal one (after and before the refinement process, respectively).

**Table 4:** Receptor interactions and binding energies of gondoic acid and ligand into the active pocket site of VEGF catalytic domain.

Cpd.	S ^a^ kcal/mole	RMSD_Refine ^b^	Amino Acid Bond	Distance Å	E (kcal/mol)
Gondoic acid	−8.362	1.67	LEU 840/H-donor	2.83	−6.30
			ASN 923/H-acceptor	3.28	−1.40
			ASN 923/H-acceptor	3.05	−2.80
Ligand	−7.97	0.9175	Asn 923/H-acceptor	3.40	−1.20
			Cys 919/H-acceptor	3.55	−1.10
			Leu 840/pi-H	3.76	−1.60
			Leu 840/pi-H	4.00	−0.50

^a^ S: The score of a compound placement inside the protein binding pocket. ^b^ RMSD_Refine: the root-mean-square deviation (RMSD) between the predicted pose and those of the crystal one (after and before the refinement process, respectively).

**Table 5:** Receptor interactions and binding energies of compounds and ligands into the active pocket site of GST catalytic domain.

Cpd.	S ^a^ kcal/mole	RMSD_Refine ^b^	Amino Acid Bond	Distance Å	E (kcal/mol)
Oleic acid	−6.764	2.023	ARG 67/H-acceptor	3.08	−3.80
3″(1‴-*O*-*β*-D-glucopyranosyl)-sucrose	−7.24	1.769	Glu 65/H-donor	3.00	−1.50
			SER 10/H-acceptor	3.19	−0.60
			Arg 67/H-acceptor	3.08	−1.10
			Ser 66/H-acceptor	3.27	−1.20
Ligand	−5.945	1.405	Glu 65/H-donor	3.03	−1.40
			Ile 53/H-donor	3.04	−3.40
			Arg 67/H-acceptor	2.85	−8.30
			Ser 66/H-acceptor	2.89	−3.60
			Arg 67/Ionic	2.85	−5.50
			Glu 65/Ionic	3.03	−4.30

^a^ S: The score of a compound placement inside the protein binding pocket. ^b^ RMSD_Refine: the root-mean-square deviation (RMSD) between the predicted pose and those of the crystal one (after and before the refinement process, respectively).

## Data Availability

Not applicable.

## Supplementary Materials

The following supporting information can be downloaded at: https://www.mdpi.com/article/10.3390/antibiotics12010043/s1, Figure S1: GC/MS spectrum for Cocos nucifera seed oil; Figure S2: 1H NMR spectrum of compound 18 measured in CD3OD-d4 at 400 MHz; Figure S3: DEPT-Q NMR spectrum of compound 18 measured in CD3OD-d4 at 100 MHz; Figure S4: 1H NMR spectrum of compound 19 measured in DMSO-d6 at 400 MHz; Figure S5: DEPT-Q NMR spectrum of compound 19 measured in DMSO-d6 at 100 MHz; Figure S6: 1H NMR spectrum of compound 20 measured in CDCL3-d at 400 MHz; Figure S7: DEPT-Q NMR spectrum of compound 20 measured in CDCL3-d at 100 MHz; Table S1: Gene primers sequences; Table S2: Relative gene expression in skin tissue of the tested rabbits; Table S3: Coconut seed extract/oil scavenging activity of H_2_O_2_ at different concentration; Table S4: Superoxide radical scavenging activity of coconut oil at many concentration; Table S5: Receptor binding energies of the compounds detected in coconut seed extract and the ligand into the active pocket site of IL-1β catalytic domain; Table S6: Receptor binding energies of compounds detected in coconut seed extract and ligand into the active pocket site of IL-6 catalytic domain; Table S7: Receptor binding energies of compounds detected in coconut seed extract and ligand into the active pocket site of VEGF catalytic domain; Table S8: Receptor binding energies of compounds detected in coconut seed extract and ligand into the active pocket site of GST catalytic domain, References [74,80,81,82,83] are cited in the supplementary materials.
