# Supplementary material for: Scabicidal Potential of Coconut Seed Extract in Rabbits via Downregulating Inflammatory/Immune Cross Talk: A Comprehensive Phytochemical/GC-MS and In Silico Proof
Source: Antibiotics (Basel). 2022 Dec 27;12(1):43. doi: 10.3390/antibiotics12010043 (PMC9854674; doi:10.3390/antibiotics12010043)
Supplement: Supplementary file 1 [file antibiotics-12-00043-s001.zip › antibiotics-1994316-SI.pdf]

antibiotics

SUPPLEMENTARY DATA

Scabicidal Potential of Coconut Seed Extract in Rabbits via Downregulating Inflammatory/Immune Cross Talk: A Comprehensive Phytochemical/GC-MS and In Silico Proof

Eman Maher Zahran 1 , Nehad M. Reda Abdel-Maqsoud 2,3, Omar. Y. Tammam 4, Islam M. Abdel-Rahman 5 , Mahmoud A. Elrehany 4, Hussain T. Bakhsh 6 , Faisal H. Altemani 7 , Naseh A. Algehainy 7 , Mubarak A. Alzubaidi 8 , Usama Ramadan Abdelmohsen 1,9,* and Abeer H. Elmaidomy 10

1 Department of Pharmacognosy, Faculty of Pharmacy, Deraya University, Minia 61111, Egypt
2 Department of Pathology, Faculty of Pharmacy, Deraya University, Minia 61111, Egypt
3 Department of Pathology, Faculty of Medicine, Minia University, Minia 61519, Egypt
4 Department of Biochemistry, Faculty of Pharmacy, Deraya University, Minia 61111, Egypt
5 Department of Pharmaceutical Chemistry, Faculty of Pharmacy, Deraya University, Minia 61111, Egypt
6 Department of Pharmacy Practice, Faculty of Pharmacy, King Abdulaziz University, Jeddah 21589, Saudi Arabia
7 Department of Medical Laboratory Technology, Faculty of Applied Medical Sciences, University of Tabuk, Tabuk 71491, Saudi Arabia
8 Department of Biological Sciences, Faculty of Science, King Abdulaziz University, Jeddah 21589, Saudi Arabia
9 Department of Pharmacognosy, Faculty of Pharmacy, Minia University, Minia 61519, Egypt
10 Department of Pharmacognosy, Faculty of Pharmacy, Beni-Suef University, Beni-Suef 62514, Egypt
* Correspondence: usama.ramadan@mu.edu.eg

Materials and methods

Seeds Collection

C. nucifera seeds were collected in 2021 from India and was kindly identified by Dr. Abd El-Halim A. Mohammed of the Horticultural Research Institute, Department of Flora and Phytotaxonomy Research, Dokki, Cairo, Egypt. A voucher specimen (2021-BuPD 81) was deposited at the Department of Pharmacognosy, Faculty of Pharmacy, Beni-Suef University, Egypt.

Chemicals and Reagents

Solvents used in this work as *n*-hexane (*n*-hex., boiling point b.p. 60–80 °C), dichloromethane (DCM), ethyl acetate (EtOAC), *n*-butanol (*n*-But.), methanol (MeOH), ethanol (EtOH), sulphuric acid, and sodium bicarbonate was purchased from El-Nasr Company for Pharmaceuticals and Chemicals, Egypt, which had been distilled before use. Deuterated solvents used for spectroscopic analyses were purchased from Sigma-Aldrich (Saint Louis, Missouri, USA), including dimethyl sulfoxide (DMSO-d_6), and methanol-d_4 (CD_3OD-d_4). Column chromatography (CC) was performed using silica gel 60 (63–200 µm, E. Merck, Sigma-Aldrich), while silica gel GF254 for Thin-layer chromatography (TLC) (El-Nasr Company for Pharmaceuticals and Chemicals, Egypt) was employed for vacuum liquid chromatography (VLC). Thin-layer chromatography (TLC) was carried out using pre-coated silica gel 60 GF254 plates (E. Merck, Darmstadt, Germany; 20 × 20 cm, 0.25 mm

in thickness). Spots were visualized by spraying with *para*-anisaldehyde (PAA) reagent (85 : 5 : 10 : 0.5 absolute EtOH : sulfuric acid :glacial acetic acid: *para*-anisaldehyde), followed by heating at 110 °C [1]. For the biological study, all the kits were produced by Biosystems SA Costa Brava 30, Barcelona (Spain), and DiaSys Diagnostic Systems GmbH, Germany.

Spectral Analyses

Proton ^{1}H and Distortionless Enhancement by Polarization Transfer-Q (DEPT-Q) ^{13}C NMR spectra were recorded at 400 and 100 MHz, respectively. Tetramethylsilane (TMS) was used as an internal standard in dimethyl sulfoxide (DMSO-d_6), and methanol-d_4 (CD_3OD-d_4), using the residual solvent peak (δ_H = 2.50 and δ_C = 39.5), and (δ_H = 3.34, 4.78 and δ_C = 49.9) as references, respectively. Measurements were performed on a Bruker Advance III 400 MHz with BBFO Smart Probe and a Bruker 400 MHz EON Nitrogen-Free Magnet (Bruker AG, Billerica, MA, USA). Carbon multiplicities were determined using a DEPT-Q experiment. HRESIMS data were obtained using an Acquity Ultra Performance Liquid Chromatography system coupled to a Synapt G2 HDMS quadrupole time-of-flight hybrid mass spectrometer (Waters, Milford, MA, USA).

Extraction and fractionation of Cocos nucifera seeds

C. nucifera seeds (3kg) was collected and air-dried in the shade for one month. After drying, the seeds were finely powdered using an OC-60B/60B grinding machine (60–120 mesh, Henan, Mainland China). The finely powdered seeds were extracted by maceration using 70% ethanol (5 L, 3×, seven days each) at room temperature, and concentrated under vacuum at 45 °C using a rotary evaporator (Buchi Rotavapor R-300, Cole-Parmer, Vernon Hills, IL, USA) to afford 300 g crude extract. The dry extract was suspended in 100 mL distilled water (H_2O), and successively portioned with solvents of different polarities (*n*-Hex., DCM, EtOAC, and *n*-but.). The organic phase in each step separately evaporated under reduced pressure to afford the corresponding fractions I (50.0 g), II (1.0 g), III (3.0 g) and IV (10.0 g), respectively, while the remaining mother liquor was then concentrated down to give the aqueous fraction (V). All resulting fractions were kept at 4 °C for biological and phytochemical investigations.

Preparation of Fatty Acids Methyl Esters

Methylation for oil isolated from seeds (*n*-hexane fraction) was done using concentrated sulphuric acid to obtain fatty acids methyl esters. Five (5) mg of fractions I was suspended in 1 mL *n*-Hex., prior to derivatization [2]. Then, 2 mLs of methanoic sulphuric acids (1% v/v) was added in vials and sealed. The sample was heated in a stopper tube at a temperature of 50°C overnight for 16 h., to speed up the reaction. This was followed by addition of 2 mL water containing sodium bicarbonate (2%: w/v) to neutralize the acids. Extractions of product were done by addition of hexane (2×5m). Evaporation to remove acids was done locally in air conditioned at room temperature for 48 h.

GC-MS Analysis

The recovered fatty acids methyl esters extract were subjected separately to chromatographic analysis using gas chromatography-mass spectrometry (GC/MS) [3]. The GC-MS instrument stands with the following specifications, Instrument: a TRACE GC Ultra Gas Chromatographs (THERMO Scientific Corp., USA), coupled with a thermo mass spectrometer detector (ISQ Single Quadrupole Mass Spectrometer). The GC-MS system was equipped with a TR-5 MS column (30 m x 0.32 mm i.d., 0.25 μm film thickness). Analyses were carried out using helium as carrier gas at a flow rate of 1.0 mL/min and a split ratio of 1:10 using the following temperature program: 60˚C for 1min; rising at 4.0˚C/min to 240˚C and held for 1min. The injector and detector were held at 210˚C. Diluted samples (1:10 hexane, v/v) of 1μL of the mixtures were always injected. Mass spectra were obtained

by electron ionization (EI) at 70 eV, using a spectral range of m/z 40-450. The identification of the chemical constituents of the essential oil was de-convoluted using AMDIS software (www.amdis.net) and identified by its retention indices (relative to n-alkanes C8-C22), mass spectrum matching to (authentic standards (when available)., Wiley spectral library collection and NSIT library database).

Isolation and Purification of Compounds from seeds extract

n-Hex., fraction (2 g) was subjected to normal vacuum liquid chromatography (VLC) fractionation using silica gel GF_{254} (column 6 × 30 cm, 50 g). Elution was performed using *n*-hex. : EtOAC gradient mixtures in order of increasing polarities (0, 5, 10, 15, 20, 25, 30, 35, 40, 45, 50, 60, 80 and 100%, 500ml each). The effluents from the column were collected in fractions (100 mL each); and each collected fraction was concentrated and monitored by TLC using the system *n*-hex. : EtOAC 8 : 2 and PAA reagent. Similar fractions were grouped and concentrated under reduced pressure to provide two sub-fractions (I_1–I_2). Subfraction I_1 (1.0 g) was further fractionated on silica gel 60 (100 × 1 cm, 50 g). Elution was performed using *n*-hex. : EtOAC gradient mixtures in the order of increasing polarities (0, 1, 2, 3,4,5,6,7,8,9 and 10%, 1 L each, FR 3 mL min^{-1}), to afford compound **20** (20 mg).

n-But. (6.0 g) was subjected to normal VLC fractionation on a silica gel (column 6 × 30 cm, 50 g). Elution was performed using DCM : MeOH gradient mixtures in the order of increasing polarities (0, 5, 10, 15, 20, 25, 30, 35, 40, 45, 50, 60, 80 and 100%, 1 L each). The effluents were collected in fractions (100 mL each); each fraction was concentrated and monitored by TLC using the system DCM : MeOH 8 : 2 and PAA reagent. Similar fractions were grouped and concentrated under reduced pressure to provide two sub-fractions (IV_1–V_2), sub-fractions IV_1, IV_2 were separately further purified on a Sephadex LH_{20} column (0.25–0.1 mm, 100 × 0.5 cm, 100 gm) which eluted with MeOH to afford compounds **18** (30 mg), **19** (50 mg), respectively.

In vitro Assays

In Vitro Antioxidant Activity

Hydrogen Peroxide Scavenging Activity

The reaction with a defined amount of exogenously provided H_2O_2 was used to determine the hydrogen peroxide (H_2O_2) scavenging activity that reflects the anti-oxidative capacity of CSE. Colorimetric analysis was used to estimate the residual H_2O_2 [4]. In brief, 20µl of the sample was mixed with 500µl of H_2O_2 and incubated at 37°C for 10 minutes. After that, 500µl of enzyme/3, 5-dichloro-2-hydroxyl-benzensulfonate solution was added and incubated at 37°C for 5 minutes. Colorimetrically, the intensity of the colored product was measured at 510nm. Positive control was ascorbic acid. By comparing the percentages of H_2O_2 scavenging activity, the percentage of H_2O_2 scavenging activity was determined by comparing the results of the test with those of the control using the following formula:

$$\text{scavenging activity} = \frac{\text{A control} - \text{A sample}}{\text{A control}} \times 100$$

IC_{50} of each sample was calculated after performing the assay at four different concentrations using Graph pad prism 7 software.

Superoxide Radical Scavenging Activity

The superoxide anion scavenging activity was measured as described by Sreenivasan et al., 2007 [5]. The superoxide anion radicals were formed in a Tris – HCL buffer (16mM, pH8.0) containing 90µl of NBT (0.3mM), 90µl of NADH (0.936mM), 0.1ml of CSE extract (125, 250, 500, and 1000 g/mL), and 0.8 mL Tris – HCl buffer (16mM, PH8.0). The reaction was initiated by adding 0.1ml PMS solution (0.12mM) to the mixture, which was then incubated at 25°C for 5, and At 560 nm, the absorbance was measured. Ascorbic acid was

selected as a reference. The percentage inhibition was obtained by comparing the test findings to those of the control using the formula below:

$$\text{Superoxide scavenging activity} = \frac{\text{A control} - \text{A sample}}{\text{A control}} \times 100$$

IC_{50} was calculated using Graph pad prism 7 software by performing the test at four different concentrations.

2.1. Quantitative Real-Time Polymerase Chain Reaction (qRT-PCR)

Table S1. Gene primers sequences.

Gene	Forward	Reverse
**GAPDH**	GTC AAG GCT GAG AAC GGG AA	ACA AGA GAG TTG GCT GGG TG
**VEGF**	CAT CAG CCA GGG AGT CTG TG	GAG GGA GTG AAG GAG CAA CC
**IL-1**	AGC TTC TCC AGA GCC ACA AC	CCT GAC TAC CTT CAC GCA CC
**IL-6**	GCC AAG TTC AGG AGT GAC GA	AGA GCC CAT GAA ATT CCG CA
**MCP-1**	GAT CCC AAT GAG TCG GCT GG	ACA GAA GTG CTT GAG GTG GTT
**ICAM**	GGC GGC TCA GTG TCT CAT T	TTC GTT CCC AGA GCG AGT G
**IL-10**	AAC AAG AGC AAG GCA GTG GA	CTA GCC GAG TTG CCA TCC TG
**KGF**	ACA ATG TGG CCA AAA ATG GCT	AGG AGA TTT TTC CCC TGG CG
**MMP-9**	GCA GAG GAG TAC CTG TTC CG	ATT ATC CAG CTC CCC CGT CT
**TIMP-1**	CCT TCT GCA ACT CCG ACC TT	GTA CCC GCA GAC ACT TTC CA

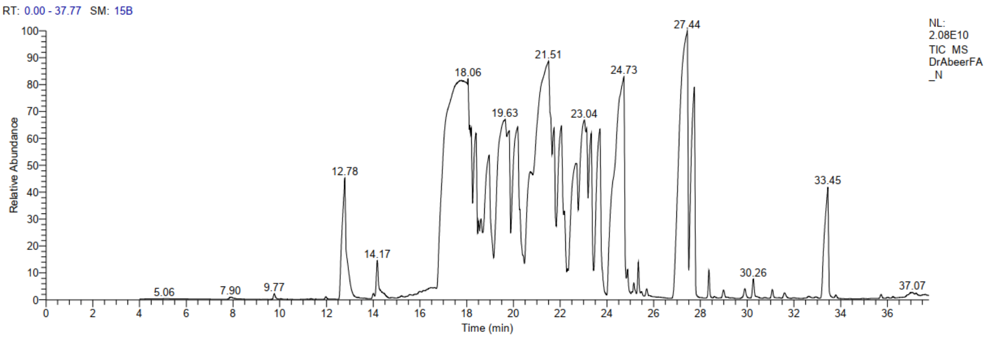

Figure S1. GC/MS spectrum for *Cocos nucifera* seed oil.

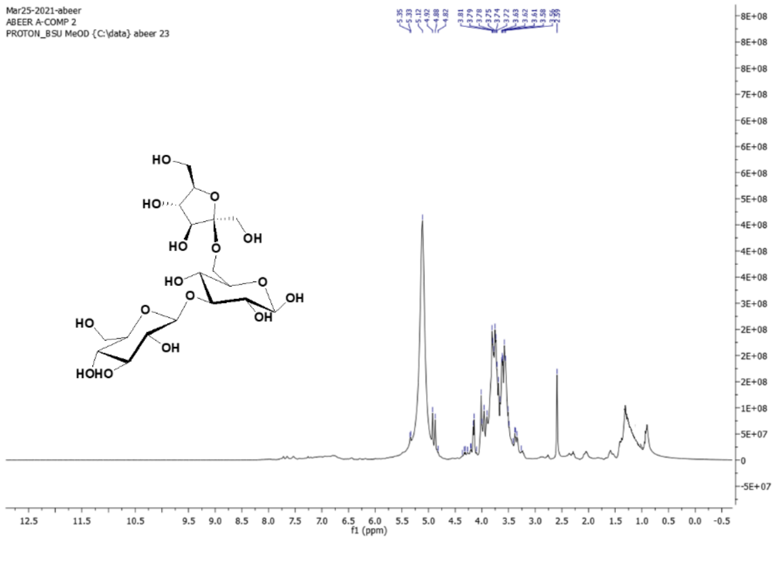

Figure S2. 1H NMR spectrum of compound **18** measured in CD_3OD-*d_4* at 400 MHz.

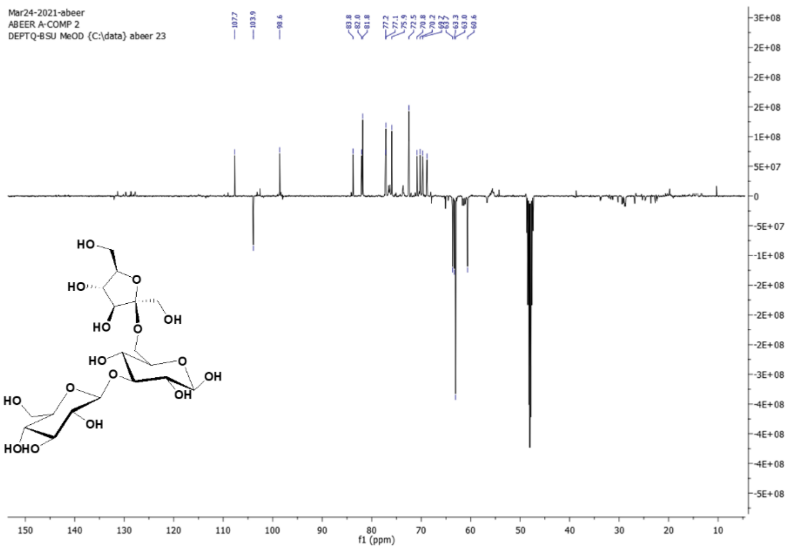

Figure S3. DEPT-Q NMR spectrum of compound **18** measured in CD_3OD-d_4 at 100 MHz.

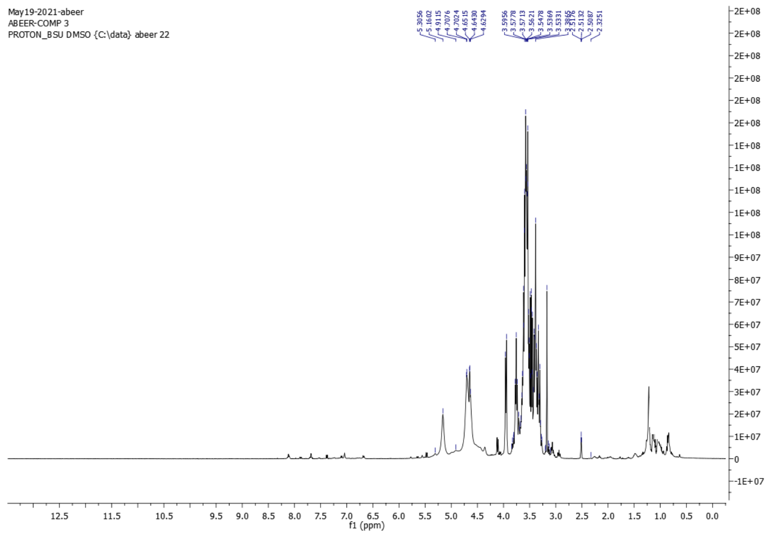

Figure S4. 1H NMR spectrum of compound **19** measured in DMSO-d_6 at 400 MHz.

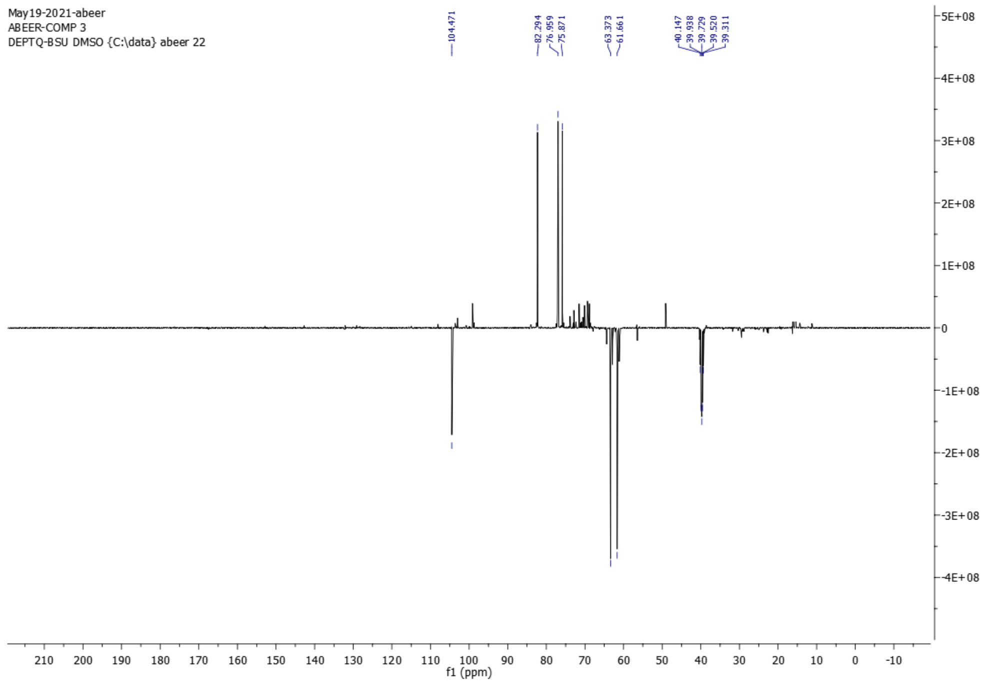

Figure S5. DEPT-Q NMR spectrum of compound **19** measured in DMSO-d_6 at 100 MHz.

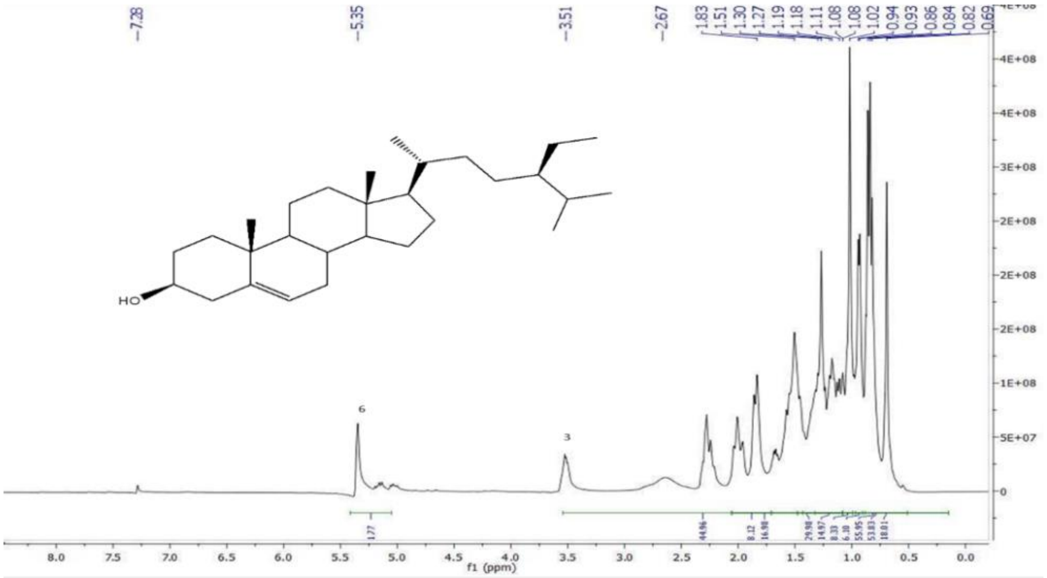

Figure S6. 1H NMR spectrum of compound **20** measured in $CDCL_3$-*d* at 400 MHz.

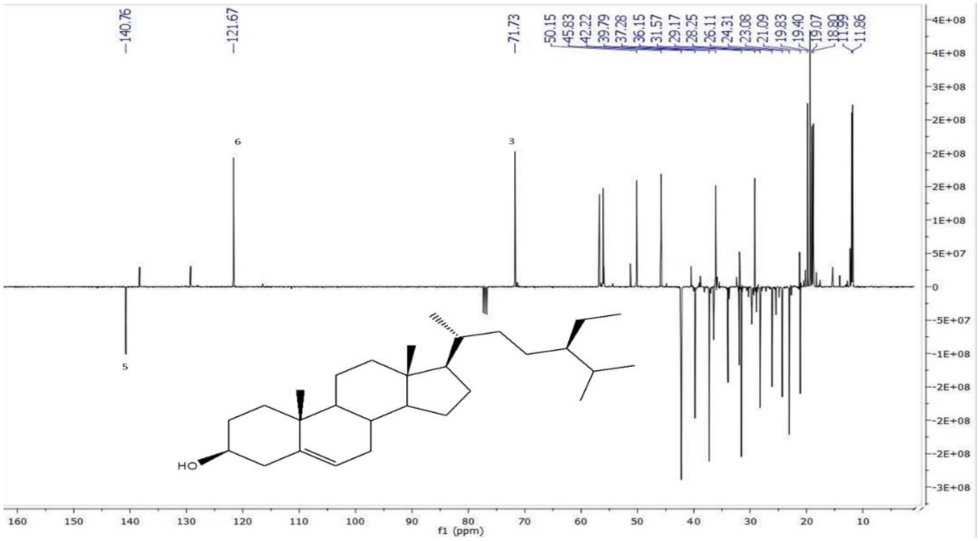

Figure S7. DEPT-Q NMR spectrum of compound **20** measured in $CDCL_3$-*d* at 100 MHz.

2.2. Q-PCR analysis

Table S2. Relative gene expression in skin tissue of the tested rabbits.

	Scabies		Market		Coconut seed extract/oil	
	**Mean**	**S.D.**	**Mean**	**S.D.**	**Mean**	**S.D.**
**VEGF**	4.82	0.21	6.25	0.132	5.47	0.14
**TIMP-1**	7.83	0.154	3.51	0.139	4.3	0.152
**mmp-9**	5.45	0.132	2.43	0.145	1.68	0.14
**MCP-1**	5.82	0.15	2.72	0.157	1.9	0.12
**KGF**	0.86	0.15	1.62	0.157	2.5	0.12
**IL-6**	3.76	0.326	1.39	0.134	1.25	0.21
**IL-10**	7.68	0.07	4.1	0.126	3	0.18
**IL-1**	5.41	0.254	2.1	0.157	2.5	0.32
**I-CAM**	0.82	0.326	3.02	0.134	2.47	0.21

Table S3. Coconut seed extract/oil scavenging activity of H_2O_2 at different concentration.

	1000 µg/mL		500 µg/mL		250 µg/mL		125 µg/mL	
	**Mean**	**S.D.**	**Mean**	**S.D.**	**Mean**	**S.D.**	**Mean**	**S.D.**
**Ascorbic acid**	81.25	1.121	56.87	0.789	53.125	1.08	34.375	1.1
**cocconut oil**	52.5	1	46.875	0.911	40.625	1.018	24.6875	0.9

Table S4. Superoxide radical scavenging activity of coconut oil at many concentration.

	1000 µg/mL		500 µg/mL		250 µg/mL		125 µg/mL	
	**Mean**	**S.D.**	**Mean**	**S.D.**	**Mean**	**S.D.**	**Mean**	**S.D.**
**Ascorbic acid**	66.12	1.1	56.87	0.8	47.68	1.09	26.12	1.08
**cocconut oil**	52.72727	1	38.18182	0.9	29.09091	1.02	16.36364	0.89

2.3. Docking analysis

Table S5. Receptor binding energies of the compounds detected in coconut seed extract and the ligand into the active pocket site of IL-1β catalytic domain.

**NO**	**Compound**	**S_a kcal/mole**	**RMSD_Refine$_b$**
**1**	1	-3.932	1.59

**2**	2	-4.271	1.576
**3**	3	-4.114	1.363
**4**	4	-4.064	1.806
**5**	5	-3.928	1.915
**6**	6	-4.26	1.478
**7**	7	-4.329	2.024
**8**	8	-4.401	1.489
**9**	9	-4.276	2.045
**10**	10	-4.615	1.233
**11**	11	-4.266	1.501
**12**	12	-5.362	1.553
**13**	13	-5.32	1.79
**14**	14	-5.189	1.917
**15**	15	-5.103	1.132
**16**	16	-5.817	1.684
**17**	17	-5.158	1.696
**18**	18	-5.115	1.9642
**19**	19	-3.775	1.29
**20**	20	-5.042	1.801
#	ligand	-5.87	1.311

Table S6. Receptor binding energies of compounds detected in coconut seed extract and ligand into the active pocket site of IL-6 catalytic domain.

NO	Compound	S_a kcal/mole	RMSD_Refine$_b$
**1**	1	-3.962	0.887
**2**	2	-4.1586	1.093
**3**	3	-4.054	1.74
**4**	4	-4.706	0.995
**5**	5	-4.098	1.867
**6**	6	-4.188	1.88
**7**	7	-4.1409	2.015
**8**	8	-4.32	1.822
**9**	9	-4.144	1.91
**10**	10	-4.0325	1.898
**11**	11	-4.264	1.574
**12**	12	-4.42	1.209
**13**	13	-4.666	1.579
**14**	14	-4.533	2.236
**15**	15	-5.156	1.197
**16**	16	-5.291	1.31
**17**	17	-5.308	2.334
**18**	18	-5.1154	1.188
**19**	19	-4.091	1.682
**20**	20	-4.538	1.376
#	ligand	-4.191	1.758

Table S7. Receptor binding energies of compounds detected in coconut seed extract and ligand into the active pocket site of VEGF catalytic domain.

NO	Compound	S_a kcal/mole	RMSD_Refine$_b$
**1**	1	-6.457	1.0174
**2**	2	-6.298	1.087
**3**	3	-6.747	2.04
**4**	4	-6.471	0.943
**5**	5	-5.52	0.639
**6**	6	-6.647	0.98
**7**	7	-6.838	1.707
**8**	8	-6.741	0.669
**9**	9	-7.201	1.436
**10**	10	-7.526	1.502
**11**	11	-7.111	1.941
**12**	12	-8.18	1.839
**13**	13	-8.111	1.547
**14**	14	-7.515	1.452
**15**	15	-7.949	1.716
**16**	16	-8.362	1.67
**17**	17	-8.1787	1.687
**18**	18	-5.47	1.744
**19**	19	-4.352	1.267
**20**	20	-5.009	2.45
#	ligand	-7.97	0.9175

Table S8. Receptor binding energies of compounds detected in coconut seed extract and ligand into the active pocket site of GST catalytic domain.

NO	Compound	S_a kcal/mole	RMSD_Refine$_b$
**1**	1	-4.44	1.215
**2**	2	-4.259	1.695
**3**	3	-5.124	1.517
**4**	4	-5.061	2.035
**5**	5	-4.555	1.351
**6**	6	-4.894	1.403
**7**	7	-5.665	1.888
**8**	8	-5.351	1.737
**9**	9	-5.336	0.796
**10**	10	-5.575	1.181
**11**	11	-6.103	1.695
**12**	12	-6.01	1.317
**13**	13	-6.221	1.683
**14**	14	-6.764	2.023
**15**	15	-5.558	1.797
**16**	16	-5.82	1.807
**17**	17	-6.217	1.909
**18**	18	-7.24	1.769
**19**	19	-4.045	0.96
**20**	20	-6.782	1.427
#	ligand	-5.945	1.405

Refrences

1. El-Sharawy, D.M.; Khater, S.; HM, E.; Sherif, N.H.; Hassan, H.M.; Elmaidomy, A.H. 99mTc-Luteolin: Radiolabeling, In Silico ADMET and Biological Evaluation as a Natural Tracer Tumor imaging. *Journal of Radiation Research and Applied Sciences* **2021**, *14*, 125-132.
2. Dworzanski, J.P.; Berwald, L.; Meuzelaar, H.L. Pyrolytic methylation-gas chromatography of whole bacterial cells for rapid profiling of cellular fatty acids. *Applied and environmental microbiology* **1990**, *56*, 1717-1724.
3. Elmaidomy, A.H.; Hassan, H.M.; Amin, E.; Mohamed, W.; Hetta, M.H. Premna odorata volatile oil as a new mycobacterium tuberculosis growth inhibitor for the control of tuberculosis disease. *European Journal of Medicinal Plants* **2017**, 1-11.
4. Hassan, H.; Abdel-Aziz, A. Evaluation of free radical-scavenging and anti-oxidant properties of black berry against fluoride toxicity in rats. *Food and chemical toxicology* **2010**, *48*, 1999-2004.
5. Sreenivasan, S.; Ibrahim, D.; MOHD KASSIM, M.J.N. Free radical Scavenging Activity and Total Phenolic Compounds of Gracilaria changii. *International Journal of Natural & Engineering Sciences* **2007**, *1*.
